# Increased Risk of Post-Thrombolysis Intracranial Hemorrhage in Acute Ischemic Stroke Patients with Leukoaraiosis: A Meta-Analysis

**DOI:** 10.1371/journal.pone.0153486

**Published:** 2016-04-20

**Authors:** Qianqian Lin, Zhong Li, Rui Wei, Qingfeng Lei, Yunyun Liu, Xiaodong Cai

**Affiliations:** Department of Neurology, The Sixth Affiliated Hospital of Sun Yat-Sen University, Guangzhou, China; Massachusetts General Hospital, UNITED STATES

## Abstract

**Background:**

Leukoaraiosis is common in patients with acute ischemic stroke. The results from many studies investigating the association between leukoaraiosis and intracranial hemorrhage after thrombolysis remain conflicting.

**Methods:**

A meta-analysis was performed to compare the risk of post-thrombolytic intracranial hemorrhage in patients with and without leukoaraiosis. Relevant reports were identified by searching PubMed, EmBase, Cochrane Library, and ISI Web of Science through December 2015 using a combination of subjective and random terms. Eligible studies that were original articles with a clear definition of leukoaraiosis and intracranial hemorrhage were selected and analyzed. Funnel plots, Egger’s test, and Begg’s test were conducted to assess the publication bias. Sensitivity analysis was also performed to evaluate the influence of each individual study.

**Results:**

Eleven trials that enrolled 6912 participants were included. There was a significantly increased risk for acute ischemic stroke patients with leukoaraiosis (odds ratio: 1.89, 95% confidence interval 1.51–2.37, P<0.001). Low heterogeneity and less publication bias was detected among these studies. The results of both computed tomography and magnetic resonance imaging performed on the subgroups of leukoaraiosis were significant. Furthermore, an association between leukoaraiosis and symptomatic intracranial hemorrhage was also confirmed. The odds ratios remained stable with no obvious variations on the sensitivity analysis. The limitations consisted of types of including trials and not matching some baseline variables.

**Conclusions:**

The results of this meta-analysis show that leukoaraiosis approximately doubles the incidence of intracranial hemorrhage after thrombolytic therapy. However, it does not critically affect decision making regarding thrombolysis for patients with acute ischemic stroke. Additional investigations are required.

## Introduction

Ischemic stroke is one of the major causes of human death and disabilities worldwide and carries a huge economic burden [1, 2]. Thrombolytic treatment with recombinant tissue plasminogen activator (rtPA) is an effective therapy that reduces neurological impairment and improves individual survival in patients with acute ischemic stroke [3–5]. However, intracranial hemorrhage (ICH) is a significant complication for thrombolysis [6–8]. Especially, a symptomatic ICH is closely associated with poor outcomes among those patients [9, 10]. Identification of the risk factors for intracranial hemorrhage after thrombolytic therapy has drawn great attention. In the previous studies, many clinical characteristics that were associated with increasing risk of ICH in rt-PA treated patients have been indicated including advanced age, diabetes mellitus, higher stroke severity, atrial fibrillation, cardiac diseases, renal dysfunction, oral antiplatelet drugs, decreased platelet account, elevated blood pressure at admission, early infarct signs in computed tomography (CT), and so on [11–13].

Leukoaraiosis (LA) is characterized by chronic ischemic change of the cerebral white matter, which suggests microcirculation damage and can be visualized by computed tomography or magnetic resonance imaging (MRI) [14]. It is well recognized that leukoaraiosis is more common in elderly population and is a marker of vascular risk [15–17]. Leukoaraiosis has been reported to increase ICH occurrence among patients on oral anticoagulation [18, 19]. Moreover, it is thought to heighten the negative effects of ischemic stroke and rt-PA on the blood-brain barrier on the basis of abnormalities of vascular structure [20,21]. Furthermore, studies exploring the relationship between leukoaraiosis and thrombolysis-related ICH have been reported in recent years. Nevertheless, responses on whether LA is a contributing factor for ICH in post-thrombolysis patients remain conflicting. Considering the discordance among different studies, we aimed to reach a consensus by performing a meta-analysis of various observational studies comparing the outcome of intracranial hemorrhage in acute ischemic stroke patients with and without leukoaraiosis after thrombolysis.

## Methods

### Search strategies

We systematically searched for relevant articles with no language limitation from four main databases including PubMed, EmBase, Cochrane Library, and ISI Web of Science from 1966 to December 31, 2015. Search terms were combined combination of, subjective and random words. As for Medical subjective heading (MeSH) and EmBase tree, these were “leukoaraiosis” or “white matter lesion” or “white matter hyperintensity” and “thrombolytic therapy” or “tissue plasminogen activator” or “urokinase”. Additionally, we used random terms for each subjective term that means the same as “leukoaraioses”, “Plasminogen Activator, Tissue”, “U-Plasminogen Activator” and so on. Details on the search strategies are provided in S1 Text. Moreover, we used methodology filters of controlled clinical trials and observational designs, downloaded from Countway Library of Medicine. This review was conducted according to Meta-Analysis of Observational Studies in epidemiology (MOOSE) guidelines [22] and the Preferred Reporting Items for Systematic Reviews and Meta-analyses (PRISMA) [23] (S1 Table).

### Inclusion criteria

All articles were carefully evaluated and eligible articles were included if they met the following criteria: (1) enrolling patients with acute ischemic stroke on thrombolytic therapy; (2) clearly defined leukoaraiosis by CT or MRI; (3) intracranial hemorrhage including hemorrhage transformation, symptomatic intracranial hemorrhage, and parenchymal hemorrhage as described outcomes in the study; (4) original articles.

### Exclusion criteria

The exclusion criteria were as follows: (1) systematic review, meta or pool analysis, case report, or editorial; (2) if the same data has been published more than once, we selected only, the most relevant one; (3) lack of available data for analysis.

### Data extraction, data synthesis and quality assessment

All retrieved articles were read and assessed by two independent authors, and disagreements were resolved by discussion. All studies were assessed by the Newcastle-Ottawa Scale (NOS), which was used for observational studies in meta-analysis. When the score was equal to or more than six, it was believed to be of good quality. In contrast, a score equal to or less than five was thought to represent suboptimal quality. The following information were extracted from eligible studies: first author’s name, year of publication, race, time window, radiology, leukoaraiosis grading scale, ICH definition, sample size, number of cases (leukoaraiosis), and control (no leukoaraiosis) with ICH. Total data were recorded twice to prevent transcription error. No ethical approval and patient consent were required for the reason that all analyses were conducted according to previously published studies.

### Statistical analysis

Review Manager 5.3 and STATA14.0 were applied to analyze the extracted data. Random-effectsmodel was used for statistical analysis. We calculated the odds ratio (OR) for the dichotomous data, and the 95% confidence interval was also expressed. P < 0.05 was considered to be statistically significant. Heterogeneity was assessed by χ^2^ and I^2^ value. If the P value of χ^2^ is less than 0.1, homogeneity was rejected. For I^2^ statistic, 25%, 50%, and 75% were the threshold for low, moderate, high, and very high heterogeneity. Sensitivity analysis was performed to test each individual study’s contribution to the pool results. We conducted funnel plots, Egger’s test and Begg’s test to assess publication bias.

## Results

### Literature searching

**Fig 1:**
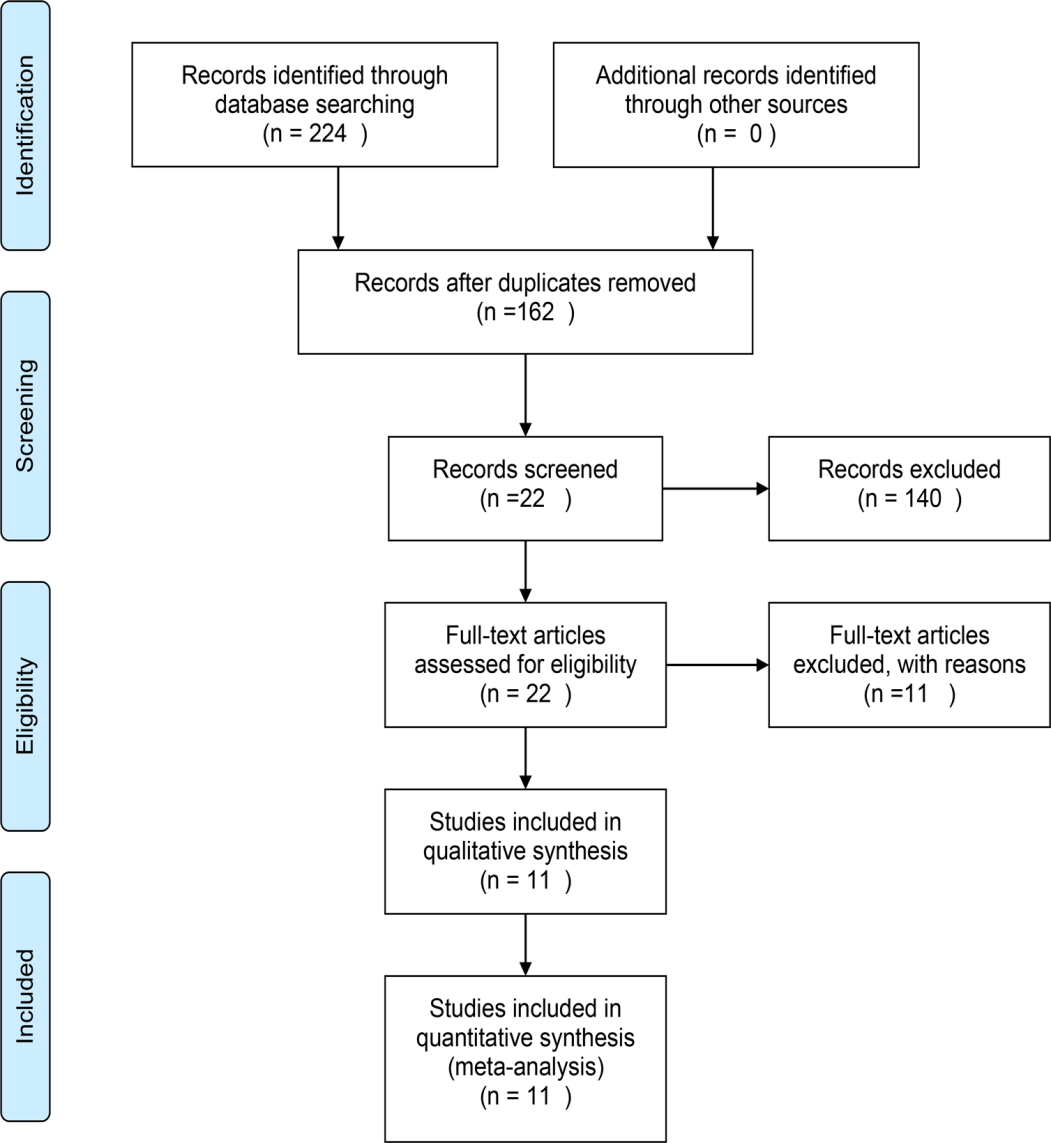
Flow chart of study selection in the meta-analysis.

A total of 224 records were identified through database searching. Of these, 162 records were retained after rejecting replications. A total of 140 articles were excluded according to the title and abstract. The reasons for the exclusion were the following: review, case report, animal experiment, no stroke, no association with leukoaraiosis, and no relevance to stroke with thrombolysis. The full-text articles were evaluated for the remaining 22 studies, and 11 were recruited in the final meta-analysis. The other 11 investigations were excluded for not meeting the inclusion criteria: five were without ICH outcome, two were based on the same population, and four were without original details for analysis. The entire selection process is shown in Fig 1. Based on NOS criteria, 10 articles were of good quality while one was of suboptimal quality (Table 1).

**Table 1:** Quality Assessment of Included Studies.

Author	Selection	Comparability	Exposure
**Tobias2006**	**2**	**1**	**3**
**V.Palumbo2007**	**3**	**1**	**3**
**Demchuk2008**	**3**	**0**	**3**
**Fiehler2009**	**3**	**0**	**3**
**Aries2010**	**3**	**1**	**3**
**Choi2011**	**3**	**1**	**3**
**Costello2012**	**3**	**0**	**3**
**Zheng2012**	**2**	**0**	**3**
**Willer2015**	**3**	**1**	**3**
**Wardlaw2015**	**3**	**0**	**3**
**Curtze2015**	**3**	**0**	**3**

### Characteristics of included research

Eleven clinical trials exploring the relationship between leukoaraiosis and post-thrombolysis ICH were included and involved total 6912 patients with acute ischemic stroke. The characteristics of each trial are shown in Table 2. There were 2884 patients with leukoaraiosis in the patient group while 4028 without LA assigned to the control group. Ten articles were published in English [24–33] and one article was written in Chinese [34]. Among these 11 trials, four investigations were conducted in Europe [24, 26, 29] and two studies were performed in Canada [31, 32]. Two trials were conducted in Asian countries [28, 34]. One was from Australia [27] and the other two studies were conducted in many different countries [25, 33]. Four studies were based on the Van Swieten Scale (VSS) [29, 31, 32, 35] or the modified Van Swieten Scale (mVSS) [27] for leukoaraiosis grading using CT scanning. Fazekas and Schmidt Scale [36] was applied in three different trials with MRI usage [28, 30, 33]. Two studies used more than one rating scales [25, 26]. Aging related white matter change (ARWMC) criterion [37] was applied in one study [24]. No grading standards were mentioned in one study [34]. Symptomatic intracranial hemorrhage (sICH) as an outcome was identified in eight groups [25–33]. However, the definition of sICH varied from clinical design-to-design. As for the remaining three studies, they were regarding intracranial hemorrhage (ICH) [34], hemorrhage transformation (HT) [24], and parenchymal hemorrhage(PH)[30].

**Table 2:** Characteristics of Included Studies.

Author	Year	Country	Drug	Method	Time	Imaging	LA grading	ICH definition
**Neumann**	**2006**	**Multicountry**	**tPA/urokinase**	**IV/IA**	**< = 6h**	**MRI**	**Fazekas and Schmidt**	**clinical deterioration within 36h = sICH**
**Palumbo**	**2007**	**Canada**	**tPA**	**IV**	**< = 3h**	**CT**	**Van Swieten scale**	**clinical deterioration within 24h = sICH**
**Demchuk**	**2008**	**Canada**	**rtPA**	**IV**	**< = 3h**	**CT**	**Van Swieten scale**	**clinical deterioration within 24h = sICH**
**Fiehler**	**2009**	**Germany**	**tPA**	**IV**	**< = 6h**	**MRI**	**Fazekas and Schmidt**	**parenchymal hematoma = PH**
**Aries**	**2010**	**Netherland**	**tPA**	**IV**	**< = 4.5h**	**CT**	**Van Swieten scale**	**NIHSS increase> = 4 within 24h = sICH**
**Choi**	**2011**	**Korea**	**tPA**	**IV/IA**	**< = 6h**	**MRI**	**Fazekas and Schmidt**	**NIHSS increase> = 4 within 24h = sICH**
**Costello**	**2012**	**Australia**	**tPA**	**IV**	**< = 4.5h**	**CT**	**modified vSS**	**NIHSS increase> = 4 within 48h = sICH**
**Zheng**	**2012**	**China**	**rtPA**	**IV**	**< = 6h**	**CT**	**/**	**hemorrhage within 24h = ICH**
**Willer**	**2015**	**Denmark**	**tPA**	**IV**	**< = 4.5h**	**CT**	**ARWMC**	**hemorrhage transformation in 36h = HT**
**Wardlaw**	**2015**	**Multicountry**	**rtPA**	**IV**	**< = 6h**	**CT/MRI**	**vSS/ Fazekas and Schmidt**	**clinical deterioration within 7d = sICH**
**Curtze**	**2015**	**Finland**	**/**	**IV**	**/**	**CT**	**More than one scales**	**NIHSS increase> = 4 within 7d = sICH**

tPA = tissue plasminogen activator, rtPA = recombinant tissue plasminogen activator, IV = intravenous, IA = intra-arterial, CT = computed tomography, MRI = magnetic resonance imaging, vSS = Van Swieten scale, ARWMC = Aging Related White Matter Change, More than one scales = Gorter scale, van Swieten scale, Blennow rating scale, the Wahlundrating scale, LA = leukoaraiosis, ICH = intracranial hemorrhage, sICH = symptomatic intracranial hemorrhage, NIHSS = National Institute of Health Stroke Scale, h = hours, d = days, / = not mention

### Outcome of meta-analysis

**Fig 2:**
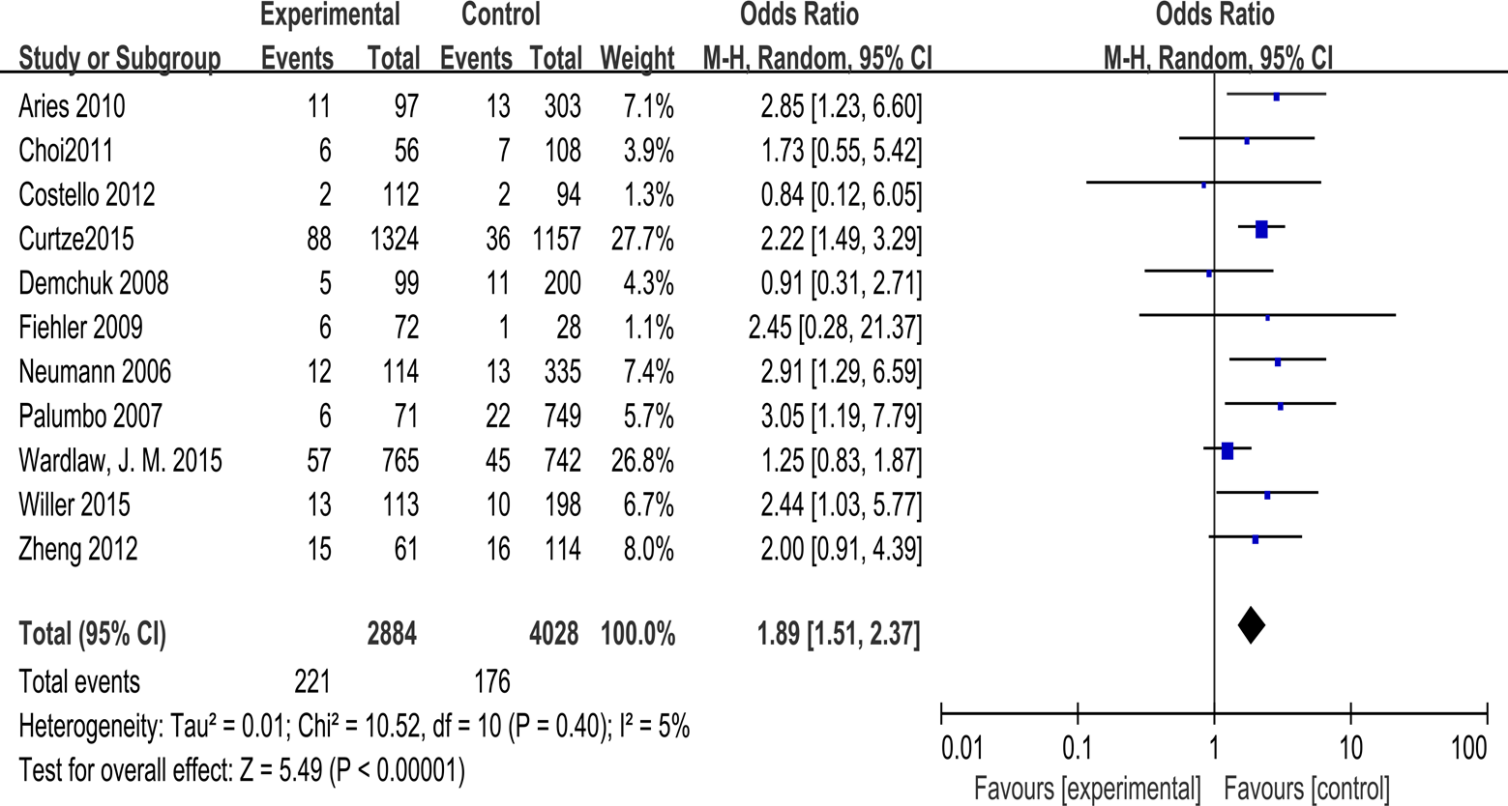
Association of leukoaraiosis with post-thrombolysis ICH in patientswith ischemic stroke.

The random-effects model was applied to pool data because there were different definitions of outcome and baseline characteristics of patients among trials. Acute ischemic stroke patients with leukoaraiosis are at a significantly higher risk of intracranial hemorrhage after being treated for thrombolysis (odds ratio: 1.89, 95% confidence interval 1.51–2.37, P < 0.001) with low heterogeneity (χ^2^ = 10.52, df = 10 [P = 0.40]; I^2^ = 5%) (Fig 2). While paying attention to the outcome as symptomatic ICH, the results also revealed a significantly elevated risk for post-thrombolysis patients (odds ratio: 1.88, 95% confidence interval 1.37–2.58, P < 0.001) with moderate heterogeneity (χ^2^ = 10.02, df = 7 [P = 0.19]; I^2^ = 30%) (S1 Fig). For subgroups divided by imaging, leukoaraiosis remained significantly associated with ICH using CT scan (odds ratio: 2.16, 95% confidence interval 1.64–2.86, P < 0.001) without heterogeneity (I^2^ = 0). At the same time, significant risk was demonstrated (odds ratio: 2.44, 95% confidence interval 1.30–4.61, P = 0.006) when focusing on groups in which MRI was performed, and there was no heterogeneity in this group (I^2^ = 0). As for the study that included both CT and MRI for leukoaraosis grading, there was no significant relation between LA and bleeding (odds ratio: 1.25, 95% confidence interval 0.83–1.87, P = 0.28>0.05). However, only one trial of this type was included, and the limited data should be interpreted with care (S2 Fig).

### Publication bias evaluation

**Fig 3:**
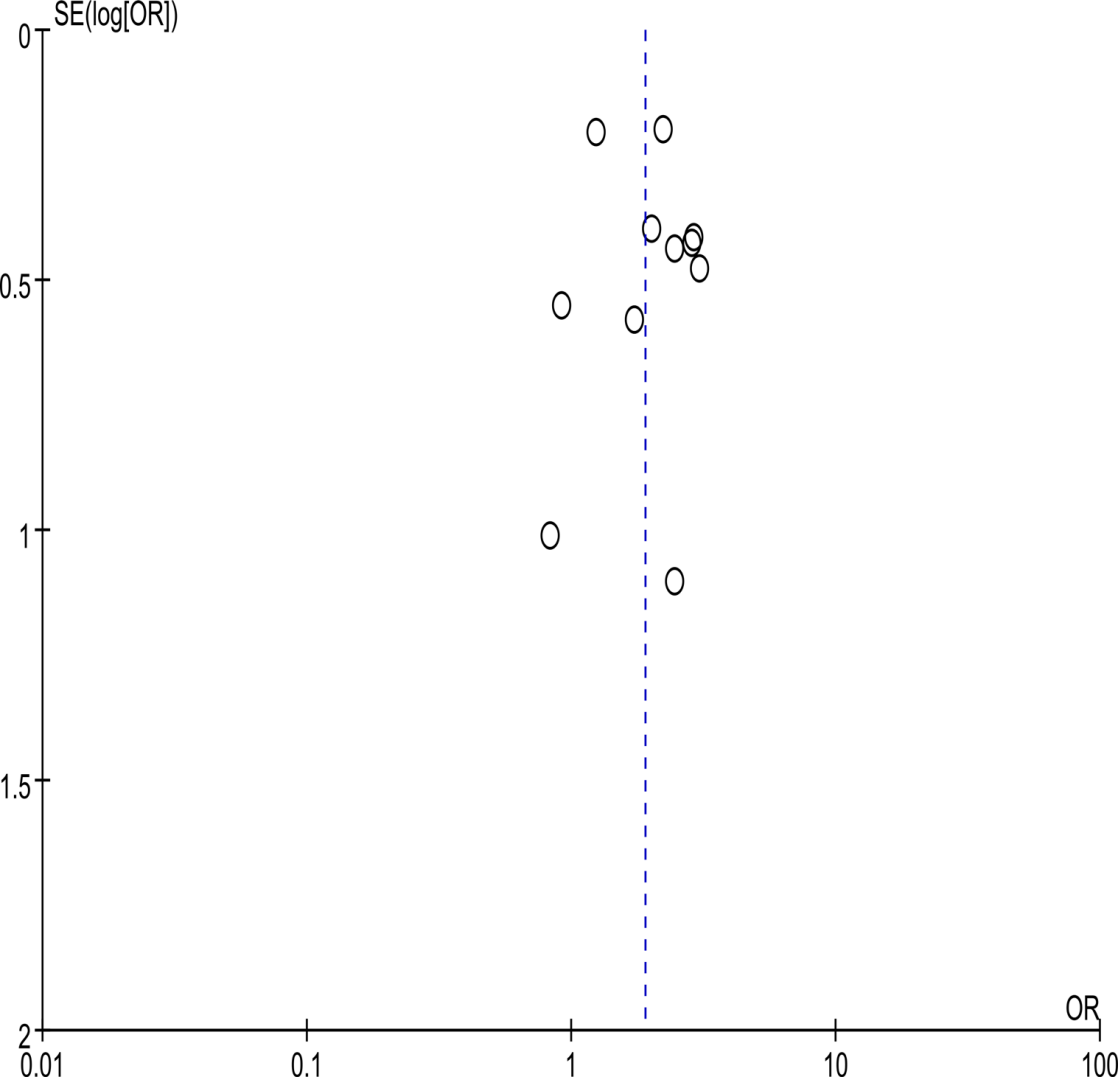
Funnel plot of the publication bias in the meta-analysis.

Overall, no significant publication bias was detected by using Begg’s test (P = 0.640) and Egger’s test (P = 0.542). Moreover, the shape of the funnel plot did not indicate any obvious asymmetry on visual inspection (Fig 3).

### Sensitivity analysis

**Fig 4:**
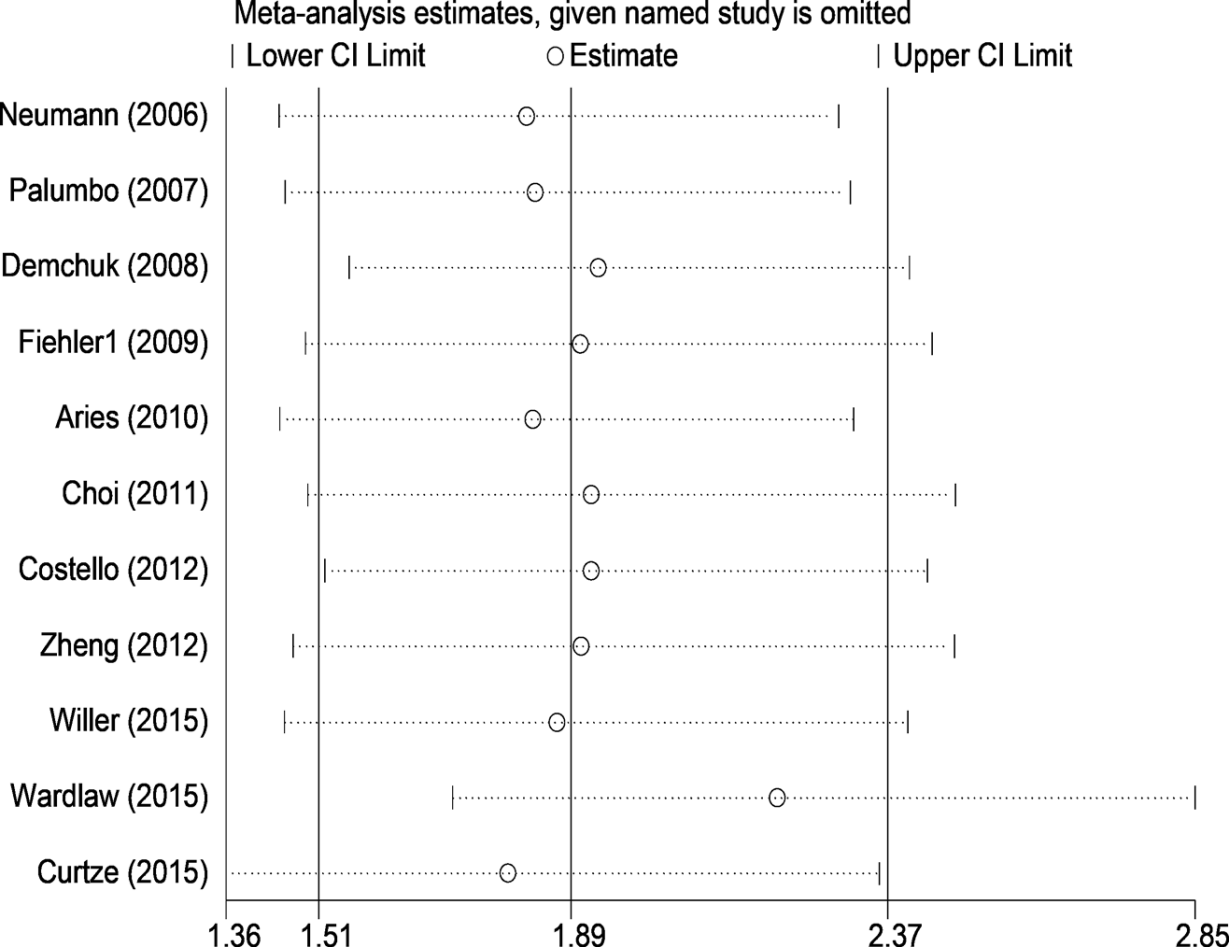
Forest plot of sensitivity analysis in the meta-analysis.

On sensitivity analysis, we subsequently omitted each individual study to recalculate the odds ratio. The re-evaluated odds ratios ranged from 1.80, on excluding the study of Curtze et al., to2.21 on the omission of the study by Wardlaw et al., with no obvious fluctuation. All the estimated odds ratios were in the 95% confidence interval of the pool result from the total 11 trials. None of the studies had great influence on the final odds ratio. The details of results are presented in Fig 4.

## Discussion

Leukoaraiosis is a term, first defined by Hachinski et al., for the description of an abnormal appearance of subcortical white matter on a CT scan [38]. It can be easily recognized through T2-weighed MRI image [39]. It has been reported to be associated with hypertension [40], diabetes mellitus [41, 42], and cognitive impairment [43]. In addition, leukoaraiosis contributed to an enhanced risk of poor neurological recovery and hemorrhage complication of ischemic stroke in some clinical trials [18, 44, 45]. There are many different hypothetical pathologic mechanisms underlying these white matter lesions like chronic small vessel hypoperfusion, endothelial dysfunction [46], blood-brain barrier damage [20, 21, 47], and genetic factors [48, 49].

The correlation between cerebral microbleeding (CMB) and leukoaraiosis is gaining attention [50, 51]. In one study, deep microhemorrhage detected by T2*-weighed gradient echo sequence technique MRI was related to the periventricular hyperintensities[51]. Furthermore, evidences indicating CMB as a predicting factor for both primary ICH and hemorrhage transformation are abundant [52–54]. Despite the results suggesting no connection between CMB and post-thrombolysis sICH from a large clinical trial [55], it is reasonable to speculate and investigate the role of leukoaraiosis in ICH complication of patients treated with thrombolysis therapy.

In recent years, increasing number of studies is focused on the association between leukoaraiosis and ICH after thrombolysis, and the results are ambiguous. There are some explanations for these conflicting outcomes. First, different radiological methods were applied. On one hand, evaluation of LA is more precise with MRI than with CT. In other words, the inference of no significant association is more easily reported in a CT-based study. Nevertheless, on the other hand, CT is more widely used worldwide than MRI. Therefore, it is necessary to compare and combine results from MRI or CT based trial. Second, varying definitions and criteria of LA grading and ICH will interfere with the conclusion. Third, as for therapy, the usage of different drugs like tPA, rtPA, and urokinase and the time window and the thrombolysis route ranging from intravenous, intra-arterial, or combined, both can affect the results. Fourth, the baseline variables of included patients such as age limitations are also important causes. Hence, we performed a meta-analysis to discuss this problem and all the above-mentioned explanations can be potential sources of heterogeneity.

Our results showed an almost doubly increased risk of ICH in acute ischemic stroke patients with leukoaraiosis after thrombolysis treatment, with low heterogeneity, which is consistent with a recent pool analysis from four original articles (odds ratio: 2.0, 95% confidence interval 1.2–3.2, P = 0.005) [24] and a system review based on five studies without detailed statements (odds ratio: 2.45, 95% confidence interval 1.64–3.66, P < 0.001) [12]. Sensitivity analysis and no obvious publication bias further demonstrated the reliability of the final results. With the considerations mentioned above, the odds ratios separately derived from CT-dependent group and MRI-dependent group also significantly increased. In addition, the OR was larger in the MRI group than in the CT group. As for sICH, there was also a significantly elevated risk with moderate heterogeneity. However, our results only prove that leukoaraiosis is a risk factor of post-thrombolytic ICH and should not be misinterpreted into excluding or withholding thrombolysis in patients with leukoaraiosis. The findings from a study in 2008 suggested that it was still beneficial for patients with leukoaraiosis to be treated with thrombolysis [31]. Therefore, it cannot outweigh the benefits of thrombolytic treatment.

Our meta-analysis has many strengths. First, we used Newcastle-Ottawa Scale (NOS) to assess each individual study to ensure quality, and the majority of included studies were at a good level, although the results changed slightly when omitting the only one research, which was graded for 5 score. At the same time, recruited trials from different countries were reliable. Yet, some limitations should be noticed. Our results were based on observational studies, not randomized clinical trials, which could have brought in bias. However, it is hard to design and perform randomized trials on this topic for the reason that leukoaraiosis is the characteristics of patients.In addition, the baseline variables of each group were not comparable. The characteristic of age differed significantly between the LA and non-LA group in many studies. LA was more common in older people. Advanced age was previously reported to be a risk factor of sICH[56]. However, the conclusions from many recent studies support that age is not a potential predictor for increased ICH after stroke thrombolysis [27, 34]. Moreover, classification of LA level and time window of thrombolysis was not performed because of lack of adequate data for analysis. Further studies are needed for a complete understanding on this topic.

## Conclusion

In summary, acute ischemic stroke patients with leukoaraiosis are at an increased risk of ICH after thrombolysis. However, it should not take precedence over the advantage of thrombolysis and prevent thrombolysis treatment.

## Supporting Information

S1 Fig Subgroups of association of leukoaraiosis with post-thrombolysis ICH in patients with acute ischemic stroke based on ICH definition. (TIF)

S2 Fig Subgroups of association of leukoaraiosis with post-thrombolysis ICH in patients with acute ischemic stroke based on imaging. (TIF)

S1 Table PRISMA checklist of the meta-analysis. (DOC)

S1 Text Searching strategies in the meta-analysis. (DOCX)
