# Supplementary figures and images for: Increased Risk of Post-Thrombolysis Intracranial Hemorrhage in Acute Ischemic Stroke Patients with Leukoaraiosis: A Meta-Analysis
Source: PLoS One. 2016 Apr 20;11(4):e0153486. doi: 10.1371/journal.pone.0153486 (PMC4838243; doi:10.1371/journal.pone.0153486)

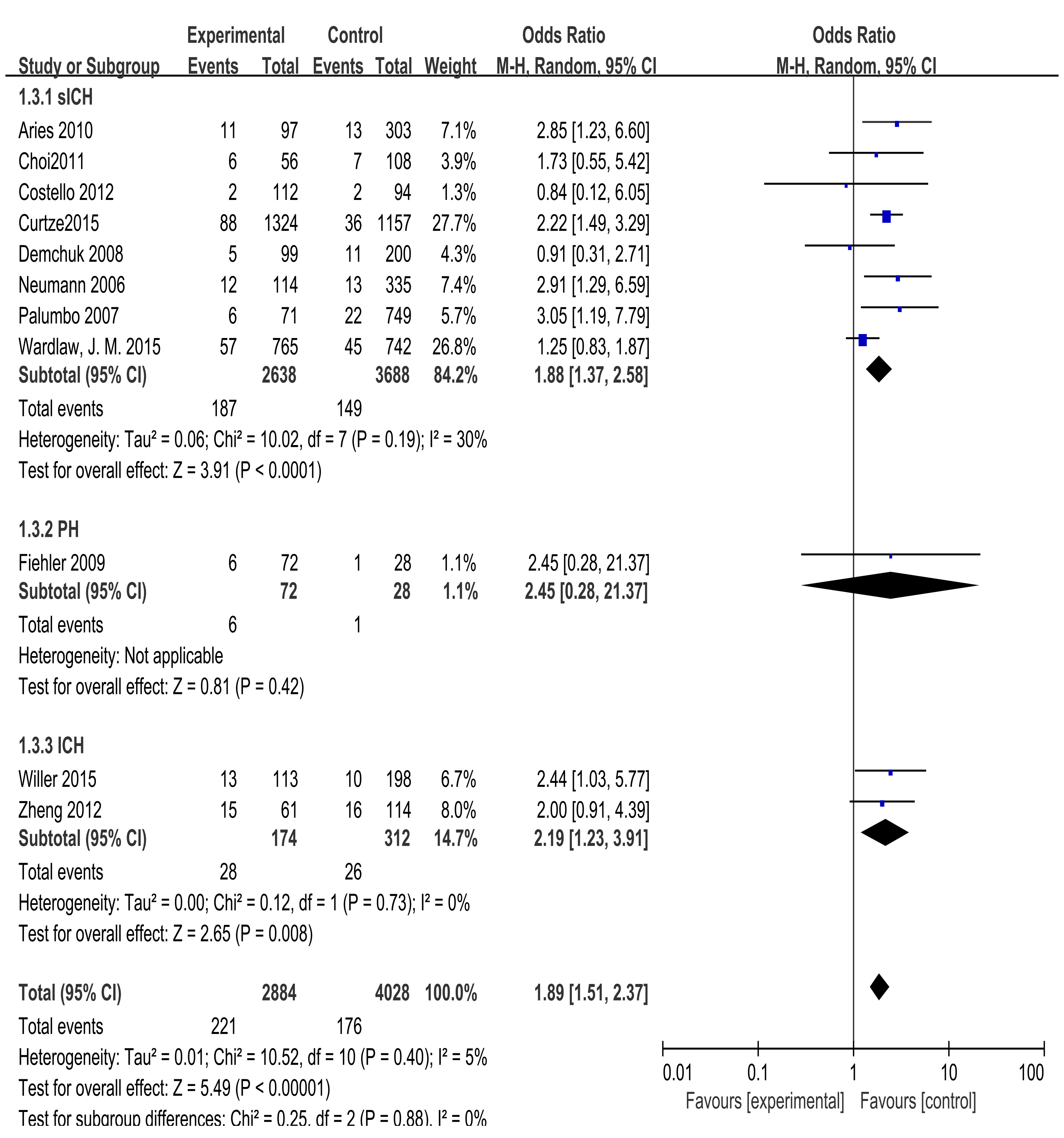

Supplement: S1 Fig — (TIF) [file pone.0153486.s001.tif]

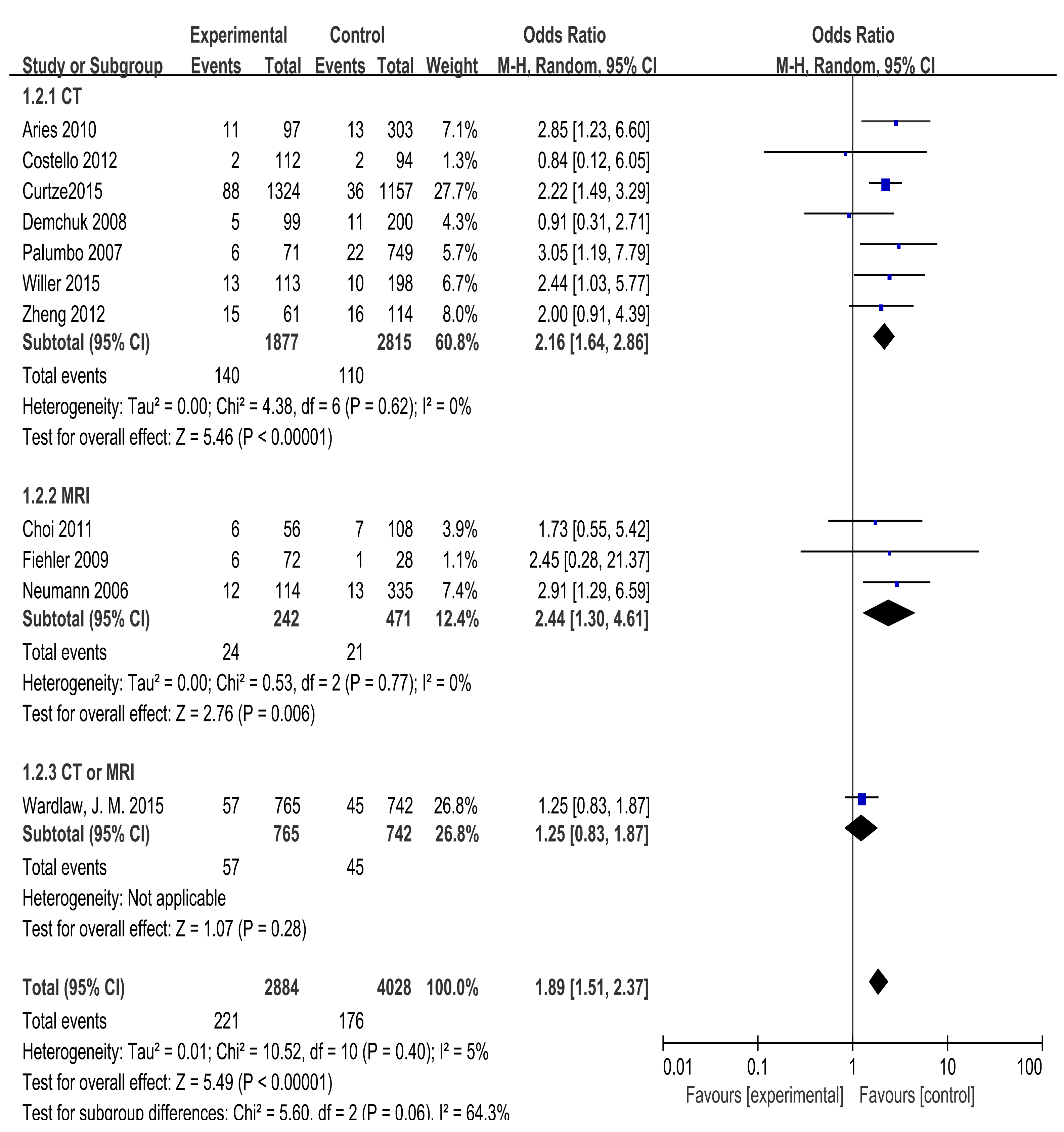

Supplement: S2 Fig — (TIF) [file pone.0153486.s002.tif]
