# Supplementary material for: Increased Risk of Post-Thrombolysis Intracranial Hemorrhage in Acute Ischemic Stroke Patients with Leukoaraiosis: A Meta-Analysis
Source: PLoS One. 2016 Apr 20;11(4):e0153486. doi: 10.1371/journal.pone.0153486 (PMC4838243; doi:10.1371/journal.pone.0153486)
Supplement: S1 Text — (DOCX) [file pone.0153486.s004.docx]

Searching strategy

**Pubmed:**

((((((((("Urokinase-Type Plasminogen Activator"[Mesh]) OR ((((((((((((Urokinase Type Plasminogen Activator) OR Plasminogen Activator, Urokinase-Type) OR U-Plasminogen Activator) OR U Plasminogen Activator) OR U-PA) OR Urinary Plasminogen Activator) OR Urokinase) OR Renokinase) OR Abbokinase) OR Kidney Plasminogen Activator) OR Single-Chain Urokinase-Type Plasminogen Activator) OR Single Chain Urokinase-Type Plasminogen Activator))) OR (((((((((((Therapeutic Thrombolysis) OR Therapeutic Thrombolyses) OR Thrombolyses, Therapeutic) OR Thrombolysis, Therapeutic) OR Therapy, Fibrinolytic) OR Fibrinolytic Therapies) OR Therapies, Fibrinolytic) OR Therapy, Thrombolytic) OR Therapies, Thrombolytic) OR Thrombolytic Therapies) OR Fibrinolytic Therapy)) OR ((((((((((((((((((((((((((Plasminogen Activator, Tissue) OR Tissue Activator D-44) OR Tissue Activator D 44) OR Tisokinase) OR Tissue-Type Plasminogen Activator) OR Tissue Type Plasminogen Activator) OR TTPA) OR T-Plasminogen Activator) OR T Plasminogen Activator) OR Alteplase) OR Plasminogen Activator, Tissue-Type) OR Plasminogen Activator, Tissue Type) OR Activase) OR Hoffmann-La Roche Brand of Alteplase) OR Hoffmann La Roche Brand of Alteplase) OR Genentech Brand of Alteplase) OR Alteplase Genentech Brand) OR Actilyse) OR Boehringer Ingelheim Brand of Alteplase) OR Lysatec rt-PA) OR Lysatec rt PA) OR Lysatec rtPA) OR Promeco Brand of Alteplase) OR Alteplase Promeco Brand)) OR "Tissue Plasminogen Activator"[Mesh]))) AND ((((leukoaraioses) OR "Leukoaraiosis"[Mesh])) OR ((white matter hyperintensity) OR white matter lesion)))) AND ((("cohort studies"[mesh] OR "case-control studies"[mesh] OR "comparative study"[pt] OR "risk factors"[mesh] OR "cohort"[tw] OR "compared"[tw] OR "groups"[tw] OR "case control"[tw] OR "multivariate"[tw])) OR ("randomized controlled trial"[pt] OR "controlled clinical trial"[pt] OR "clinical trials as topic"[mesh] OR "random allocation"[mesh] OR "double-blind method"[mesh] OR "single-blind method"[mesh] OR "clinical trial"[pt] OR "research design"[mesh:noexp] OR "comparative study"[pt] OR "evaluation studies"[pt] OR "follow-up studies"[mesh] OR "prospective studies"[mesh] OR "cross-over studies"[mesh] OR "clinical trial"[tw] OR ((singl*[tw] OR doubl*[tw] OR trebl*[tw]) AND (mask*[tw] OR blind*[tw])) OR placebo*[tw] OR random*[tw] OR "control"[tw] OR "controls"[tw] OR prospecitv*[tw] OR volunteer*[tw])) **total 42 results**

**Embase:**

#1 ‘thrombolytic therapy’ OR ‘Therapeutic Thrombolysis’ OR ‘Therapeutic Thrombolyses’ OR ‘Thrombolyses, Therapeutic’ OR ‘Thrombolysis, Therapeutic’ OR ‘Therapy, Fibrinolytic’ OR ‘Fibrinolytic Therapies’ OR ‘Therapies, Fibrinolytic’ OR ‘Therapy, Thrombolytic’ OR ‘Therapies, Thrombolytic’ OR ‘Thrombolytic Therapies’

#2 ‘fibrinolytic therapy’/exp

#3 ‘‘Plasminogen Activator, Tissue’ OR ‘Tissue Activator D-44’ OR ‘Tissue Activator D 44’ OR ‘Tisokinase’ OR ‘Tissue-Type Plasminogen Activator’ OR ‘Tissue Type Plasminogen Activator’ OR ‘TTPA’ OR ‘T-Plasminogen Activator’ OR ‘T Plasminogen Activator’ OR ‘Alteplase’ OR ‘Plasminogen Activator, Tissue-Type’ OR ‘Plasminogen Activator, Tissue Type’ OR ‘Activase’ OR ‘Hoffmann-La Roche Brand of Alteplase’ OR ‘Hoffmann La Roche Brand of Alteplase’ OR ‘Genentech Brand of Alteplase’ OR ‘Alteplase Genentech Brand’ OR ‘Actilyse’ OR ‘Boehringer Ingelheim Brand of Alteplase’ OR ‘Lysatec rt-PA’ OR ‘Lysatec rt PA’ OR ‘Lysatec rtPA’ OR ‘Promeco Brand of Alteplase’ OR ‘Alteplase Promeco Brand’

#4 ‘tissue plasminogen activator’/exp

#5 ‘Urokinase Type Plasminogen Activator’ OR ‘Plasminogen Activator, Urokinase-Type’ OR ‘U-Plasminogen Activator’ OR ‘U Plasminogen Activator’ OR ‘U-PA’ OR ‘Urinary Plasminogen Activator’ OR ‘Urokinase’ OR ‘Renokinase’ OR ‘Abbokinase’ OR ‘Kidney Plasminogen Activator’ OR ‘Single-Chain Urokinase-Type Plasminogen Activator’ OR ‘Single Chain Urokinase Type Plasminogen Activator’

#6 ‘urokinase’/exp

#7= #1 OR #2 OR #3 OR #4 OR #5 OR #6

#8 ‘leukoaraiosis’/exp

#9 ‘leukoaraioses’

#10 ‘white matter lesion’/exp

#11 ‘white matter hyperintensity’

#12=#8 OR #9 OR #10 OR #11

#13 'clinical article'/exp OR 'controlled study'/exp OR 'major clinical study'/exp OR 'prospective study'/exp OR 'cohort analysis'/exp OR 'cohort':ti,ab OR 'compared':ti,ab OR 'groups':ti,ab OR 'case control':ti,ab OR 'multivariate':ti,ab

#14=#7 AND #12 AND #13  **total 50 results**

**Web of science**

#1TOPIC: (leukoaraiosis) *OR* TOPIC: (leukoraioses)

#2 TOPIC: (white matter hyperintensity) *OR* TOPIC: (white matter lesion)

#3 = #1 OR #2

#4 TOPIC: (thrombolytic therapy) *OR* TOPIC: (Therapeutic Thrombolysis) *OR* TOPIC: (Therapeutic Thrombolyses) *OR* TOPIC: (Thrombolyses,Therapeutic) *OR* TOPIC: (Thrombolysis,Therapeutic) *OR* TOPIC: (Therapy,Fibrinolytic) *OR* TOPIC: (FibrinolyticTherapies) *OR* TOPIC: (Therapies,Fibrinolytic) *OR* TOPIC: (Therapy, Thrombolytic) *OR* TOPIC: (Therapies, Thrombolytic) *OR* TOPIC: (Thrombolytic Therapies) *OR* TOPIC: (Fibrinolytic Therapy)

#5 TOPIC: (tissue plasminogen activator) *OR* TOPIC: (PlasminogenActivator,Tissue) *OR* TOPIC: (Tissue Activator D-44) *OR* TOPIC: (Tissue Activator D 44) *OR* TOPIC: (Tisokinase) *OR* TOPIC: (Tissue-Type Plasminogen Activator) *OR* TOPIC: (Tissue Type Plasminogen Activator) *OR* TOPIC: (TTPA)*OR* TOPIC: (T Plasminogen Activator) *OR* TOPIC: (T-Plasminogen Activator) *OR* TOPIC: (Alteplase) *OR* TOPIC: (Plasminogen Activator, Tissue-Type) *OR* TOPIC: (Plasminogen Activator, Tissue Type) *OR* TOPIC: (Activase) *OR* TOPIC: (Hoffmann-La Roche Brand of Alteplase) *OR* TOPIC:(Hoffmann La Roche Brand of Alteplase) *OR* TOPIC: (Genentech Brand of Alteplase) *OR* TOPIC: (Alteplase Genentech Brand) *OR* TOPIC: (Actilyse)*OR* TOPIC: (Boehringer Ingelheim Brand of Alteplase) *OR* TOPIC: (Lysatec rt-PA) *OR* TOPIC: (Lysatec rt PA) *OR* TOPIC: (Lysatec rtPA) *OR* TOPIC:(Promeco Brand of Alteplase) *OR* TOPIC: (Alteplase Promeco Brand)

#6 TOPIC: (urokinase) *OR* TOPIC: (Urokinase Type Plasminogen Activator) *OR* TOPIC: (Plasminogen Activator, Urokinase-Type) *OR* TOPIC: (U-Plasminogen Activator) *OR* TOPIC: (U Plasminogen Activator) *OR* TOPIC: (UPA) *OR* TOPIC: (Urinary Plasminogen Activator) *OR* TOPIC: (TTPA)*OR* TOPIC: (Renokinase) *OR* TOPIC: (Abbokinase) *OR* TOPIC: (Kidney Plasminogen Activator) *OR* TOPIC: (Single-Chain Urokinase-Type Plasminogen Activator) *OR* TOPIC: (Single Chain Urokinase Type Plasminogen Activator)

#7 = #4 OR #5 OR #6

#8 = #3 and #7 **total 93 results**

**Cochrane library**

"leukoaraiosis":ti,ab,kw (Word variations have been searched) **total 39 results**

Note:

1. All searching processes were conducted with the time limitation from the earliest year to December 31 2015.
2. No language limitation among those searching strategies.
